# Efficacy of a novel intradermal *Lawsonia intracellularis* vaccine in pigs against experimental infection and under field conditions

**DOI:** 10.1186/s40813-020-00164-0

**Published:** 2020-10-01

**Authors:** A. A. C. Jacobs, F. Harks, R. Pauwels, Q. Cao, H. Holtslag, S. Pel, R. P. A. M. Segers

**Affiliations:** MSD Animal Health, Wim de Körverstraat 35, PO Box 31, 5830AA Boxmeer, The Netherlands

**Keywords:** Swine, Ileitis, Porcine proliferative enteropathy, Adenomatosis, Protection, Immunity, Vaccine

## Abstract

**Background:**

The efficacy of a novel inactivated intradermal *Lawsonia intracellularis* vaccine, Porcilis® Lawsonia ID, was evaluated in two experimental vaccination-challenge studies and under field conditions on a farm with a history of recurrent acute ileitis. In addition, the efficacy of the vaccine was compared to that of a commercially available live attenuated vaccine. The novel inactivated vaccine consists of a freeze-dried antigen fraction that is dissolved just prior to use in either the adjuvant or in Porcilis® PCV ID; an existing intradermal vaccine against porcine Circovirus type 2. In the two experimental vaccination-challenge studies, groups of 25 piglets were vaccinated once at 3 weeks of age or left unvaccinated as challenge control. Vaccines tested were Porcilis® Lawsonia ID as standalone (study 1) or in associated mixed use with Porcilis® PCV ID (study 2) and an orally administered commercially available live vaccine (study 1). The pigs were challenged with virulent *L. intracellularis* at 4 weeks (study 1) or 21 weeks (study 2) after vaccination. Post-challenge, the pigs were evaluated for clinical signs, average daily weight gain, shedding and macroscopic as well as microscopic immuno-histological ileum lesion scores. In the field study, the mortality and key performance parameters were evaluated over a period of 8 months.

**Results:**

The results of the two experimental vaccination-challenge studies showed that Porcilis® Lawsonia ID as single vaccine or in associated mixed use with Porcilis® PCV ID, induced statistically significant protection against experimental *L. intracellularis* infection, 4 weeks or 21 weeks after vaccination. This was demonstrated by lower clinical scores, improved weight gain, reduction of *L. intracellularis* shedding and reduction of macroscopic as well as microscopic ileum lesion scores when compared to the controls. The protection induced was superior to that of the commercially available live vaccine. In the field study Porcilis® Lawsonia ID was highly efficacious in reducing *L. intracellularis* associated mortality and improving key production parameters.

**Conclusion:**

The results support that this new intradermal vaccine is efficacious against *L. intracellularis* and may be used in associated mixed use with Porcilis® PCV ID.

## Background

*Lawsonia intracellularis* is the cause of Porcine Proliferative Enteropathy (PPE) also known as Porcine Intestinal Adenomatosis (PIA) or ileitis [1–4]. Clinically affected animals exhibit diarrhoea and reduced growth performance resulting in increased time to market and greater variation in size between pigs. In young adults, the infection can lead to an acute haemorrhagic form of the disease, but the bacterium also infects pigs sub-clinically without clear clinical signs, but still resulting in reduced growth performance [5].

Although both inactivated and live attenuated vaccines are being used in the pig industry against the disease [6, 7](http://www.enteric.solutions/), no intradermal vaccines have been available until now. Intradermal (ID) vaccination has the advantage of targeting antigen presenting cells in the epidermis in close proximity to skin-draining lymph nodes [8, 9]. Combined with needle-free administration, ID vaccination is also more animal friendly and prevent accidental transmission of pathogens caused by re-using needles as well as broken needles in the muscle and derived consumer products.

Recently we reported on the efficacy of a new inactivated one dose vaccine against *L. intracellularis* consisting of inactivated freeze-dried bacterial antigen that just before use is reconstituted in either its own adjuvant or in Porcilis® PCV M Hyo; an existing combination vaccine against porcine Circovirus type 2 and *Mycoplasma hyopneumoniae* [6]. A logical approach for the development of a new intradermal vaccine is to dissolve the same freeze-dried antigen fraction in an intradermal adjuvant (= Porcilis® Lawsonia ID). Since the intradermal adjuvant is the same for Porcilis® Lawsonia ID and Porcilis® PCV ID, an existing intradermal vaccine against porcine Circovirus type 2, both vaccines may be used in associated mixed use, thereby reducing the number of vaccinations and associated stress for the animals.

The aim of these studies was to assess the efficacy of the novel intradermal *L. intracellularis* vaccine in comparison with a commercially available live attenuated vaccine, against *L. intracellularis* challenge. Since vaccination against PCV2 is also required in the field, the associated mixed use of the new vaccine with Porcilis® PCV ID was also tested in a vaccination-challenge study. Besides these experimental vaccination-challenge studies, the vaccine was also tested in a field trial on a farm with PPE associated mortality.

## Methods

### Design of the experimental vaccination-challenge studies

Two studies were performed, each with a similar design (Table 1).

**Table 1:** Experimental design of vaccination-challenge studies

Study	group	number of pigs	vaccine / age / route / volume	age Lawsonia challenge
1	1	25	Law ID^a^ / 3w^b^ / ID^c^ / 0.2 ml	7w
	2	25	Live vaccine / 3w / oral / 2ml	7w
	3	25	unvaccinated control	7w
2	1	25	Law ID + PCV ID^d^ / 3w / ID / 0.2 ml	24w
	2	25	unvaccinated control	24w

^a^Porcilis® Lawsonia ID

^b^weeks

^c^intradermal in the neck

^d^associated mixed use of Porcilis® Lawsonia ID and Porcilis® PCV ID

For each of the two studies 75 or 50 piglets, from a farm known to be negative for *Mycoplasma hyopneumoniae* (M Hyo) and Porcine Reproductive & Respiratory Syndrome Virus (PRRSV) and no history of PPE, were randomly allotted to three or two groups of 25 piglets each, respectively. In each study one group was vaccinated once intradermally at 3 weeks of age with 0.2 ml Porcilis® Lawsonia ID (study 1) or with 0.2 ml Porcilis® Lawsonia ID in associated mixed use with Porcilis® PCV ID (study 2). In both studies one group was left unvaccinated and in study 1 a third group was vaccinated once orally with 2 ml of the live vaccine, also at 3 weeks of age. Groups were composed of pigs derived from at least 6 different sows. In addition, because the live vaccine may spread, these piglets were kept separated from the other groups. The pigs were challenged with *L. intracellularis* 4 weeks after vaccination (study 1), or 21 weeks after vaccination (study 2). The challenge was carried out by the oral administration of homogenized *L. intracellularis* infected intestinal mucosa. The pigs were observed daily for clinical signs of PPE and were weighed at weekly intervals throughout the 21-day post-challenge period. Faeces samples also were collected at weekly intervals throughout the 21-day post-challenge period. Serum samples were collected on the day of vaccination, the day of challenge and the day of necropsy. All pigs were euthanised and necropsied 21 days post-challenge. The intestines, in particular the ileum, were macroscopically checked for suggestive PPE lesions and Ileum samples were collected for qPCR and immunohistochemistry scoring (IHC).

### Design of the field trial

The field trial was carried out according to a negative controlled, randomised and masked design in a commercial pig herd in the Netherlands with a history of PPE associated mortality, i.e. acute ileitis which was observed predominantly close to slaughter age. The study involved 3261 pigs of which 1628 were vaccinated once in the fattening unit during a period of 8 months. Vaccinates and controls were commingled in pens. The pigs were vaccinated between 11 and 59 days after arriving in the fattening unit. As transfer to the finishing site was at an age of approximately 10 weeks, the pigs were between approximately 81 and 129 days old at vaccination. Half of the pigs were vaccinated with Porcilis® Lawsonia ID, whereas the control pigs were not vaccinated against *L. intracellularis*. With the exception of a PRRS vaccination at 6 weeks of age no further vaccines were administered. The mortality in vaccinates and controls was evaluated until slaughter at approximately 25 weeks of age. Key performance parameters, i.e. overall mortality, average daily weight gain (ADWG) and feed conversion rate were derived from the farm data management system for historic comparison. Pigs that died or were euthanised were examined post-mortem to establish the cause of death. *L. intracellularis* infection was determined by the specific signs of proliferative enteropathy and supported by immunohistochemistry (IHC). The primary efficacy parameter was the reduction in mortality associated with *L. intracellularis* infection. The total mortality was the secondary parameter. Key production parameters (mortality, average daily weight gain and feed conversion) of the finishing pigs were downloaded from the farm management system for the study period and the year before the start of the study. These data, collected on a farm-level, originated from both vaccinated and non-vaccinated pigs and also for the full finishing period and could, therefore, not be evaluated statistically but served as illustration to put the clinical results into perspective.

### Vaccines

Porcilis® Lawsonia ID lyophilizate, Solvent for Porcilis® Lawsonia ID (X-Solve®) and Porcilis® PCV ID were from MSD Animal Health. Just prior to use, Porcilis® Lawsonia ID lyophilisate was dissolved in either Solvent (study 1 and the field trial) or in Porcilis® PCV ID (study 2). Porcilis® Lawsonia ID was administered with the IDAL® (Intra Dermal Administration of Liquids, MSD Animal Health) injector, either alone or in associated mixed use with Porcilis PCV ID, as a single 0.2 ml dose to 3-week-old piglets and in the field trial to pigs 2–9 weeks after arrival at the finishing unit.

The commercially obtained live vaccine was prepared just before use (study 1) and administered to the individual piglets orally by drenching according to the manufacturer’s instruction.

### Challenge material

Challenge material was prepared from intestinal scrapings derived from *L. intracellularis* infected pigs (field cases). Pigs with clinical signs of PPE were transported to MSD-AH and necropsied. Affected parts of the intestines were scraped and the mucosa stored at − 70 °C until use. The scraped mucosa was used for challenge only after immuno-histological confirmation of the *L. intracellularis* infection and if there was no indication that other pathogens were involved as judged by post-mortem and histological examination.

Just prior to challenge portions of 500 g infected mucosa were thawed and mixed with 500 ml 0.04 M isotonic PBS. This mixture was homogenised in an omnimixer for 1 min at 16,000 rpm on ice. Each pig was orally challenged with 20 ml challenge material.

Different batches of challenge material derived from different sources were used in each of the two vaccination-challenge studies, but within a study all pigs were challenged with the same batch of challenge material.

### Post-challenge clinical observations

All pigs were clinically observed for signs of *L. intracellularis* infection just prior to challenge, and on a daily basis after challenge. The following scoring system was used: 0 = normal, 1 = mild diarrhoea (soft and shaped like peanut butter), 2 = moderate diarrhoea (loose like mush or yogurt), 3 = severe diarrhoea (watery and/or with incorporation of blood); all other abnormalities were described.

Animals that died or were euthanised because of a severe *L. intracellularis* infection were assigned a score of 3 for each of the remaining observation points to allow the magnitude of the clinical effect to be appropriately recognised in the analysis. In this challenge model clinical signs become apparent in the third week after challenge. The daily clinical scores 13 to 21 days post-challenge were added up and averaged by group.

### Performance / weighing

The pigs were weighed 1 day before challenge, and on days 6, 13 and 20 after challenge. In this challenge model the negative effect on growth becomes apparent in the third week after challenge. The ADWG in the third week after challenge (days 13 to 20) was calculated for each individual animal and averaged by group.

### Post-mortem examination

Three weeks after challenge the pigs were euthanised by electric stunning followed by bleeding and a post-mortem examination was carried out. During necropsy the intestines, in particular the ileum (i.e. the distal 50 cm of the small intestine), were examined for lesions indicative of PPE. A faecal sample (from the rectum) and an ileum sample (5 cm above the ileo-caecal junction) were collected from each animal for testing in a *L. intracellularis* specific qPCR. In addition, an ileum sample was collected and fixed in 4% buffered formalin and then further processed into slides. These slides were stained with Haematoxylin-Eosin (HE stain) and with an immunohistochemical stain using an anti- *L. intracellularis* monoclonal antibody (IHC stain) and were examined microscopically. The monoclonal antibody used for the IHC was an in-house developed monoclonal that recognises a surface carbohydrate antigen of *L. intracellularis*.

The ileum mucosa was macroscopically scored using the following scoring system: 0 = normal, 0.5 = slight, 1 = mild, 2 = moderate, 3 = severe thickening and/or reddening; 4 = severe thickening and/or reddening with fibrin and/or necrosis; other abnormalities were described. In addition, the percentage of the ileum affected was estimated as follows: the length of the affected part of the ileum was divided by the length of the ileum and multiplied by 100. The total ileum lesion score was calculated by multiplication of the ileum mucosa score and the percentage of ileum affected. The average total ileum lesion score was calculated for each treatment group.

The histological scoring was performed using the following scoring system for HE stain: 0 = normal tissue (no lesions), 0.5 = very mild (isolated minimal lesion), 1 = mild (isolated lesions), 2 = moderate (multifocal lesions), 3 = severe (coalescing to diffuse lesions) and for the IHC stain: 0 = no staining, 0.5 = very little focal staining, 1 = small amounts of stained bacteria covering < 30% of the mucosa surface, 2 = moderate amount of stained bacteria covering 30–50% of the mucosa surface, 3 = large amount of (intensely) stained bacteria covering > 50% of the mucosa. The total histological score was calculated by multiplication of the HE score and the IHC score. The average total histological score was calculated for each group.

### L. intracellularis serology

Serum samples were tested in an in-house *L. intracellularis* antibody ELISA. For preparation of the coating antigen *L. intracellularis* was cultured on McCoy cells, harvested by centrifugation, suspended in 40 mM PBS (40x concentrated) and then sonicated to release the antigen. Just before coating the antigen was diluted 500x in 40 mM PBS and the wells of a microtiter plate were filled with 100 μl portions and incubated for 16-18 h at 37 °C. After coating, the plates were washed and serial three-fold dilutions of the test sera were made. Following incubation and subsequent washing, the bound antibodies were quantified by using an anti-pig conjugate and a TMB substrate. Titres were expressed in log_2_. In this test, titres < 3.9 are considered negative. For calculation purposes < 3.9 was replaced by 2.9. The intermediate precision and repeatability for this ELISA were established as 0.25 log_2_ and 0.14 log_2_, respectively.

### PCV serology

In study 2, sera were tested in an in-house PCV2 blocking ELISA as described previously [10]. Serially four-fold diluted serum samples were incubated on microtitre plates coated with baculovirus expressed PCV2 ORF2 antigen. After removing the sera, all wells were incubated with a fixed amount of biotin-labelled PCV2-specific monoclonal antibody. Bound monoclonal antibody was incubated with peroxidase-conjugated streptavidin followed by chromophoric detection. Titres were expressed in log_2_. In this test titres < 2.0 are considered negative. For calculation purposes < 2.0 was replaced by 1.0.

### L. intracellularis PCR

DNA was isolated from 0.2 g faeces and/or mucosa samples using the MagNA Pure 96 robot. Before DNA isolation the faeces and mucosa samples were homogenised. To homogenise faeces, 550 μl STAR buffer was added and subsequently vortexed. To 250 μl homogenate 250 μl lysis buffer and 50 μl proteinase K were added and incubated for 10 min. at 65 °C followed by 10 min. at 95 °C. To homogenise mucosa, the material was transferred to a MagNA Lyser green bead tube, 800 μl lysis buffer was added and subsequently homogenised in the MagNA lyser for 30 s. at 7000 rpm. From the homogenised faeces and/or mucosa, 200 μl was processed in the MagNA Pure 96 with the kit DNA/Viral NA SV and protocol Pathogen Universal 200. The MagNA Pure96, MagNA Lyzer, and buffers were from Roche. An in-house quantitative PCR on the AspA gene of *L. intracellularis* was performed using the Kapa probe fast universal qPCR kit mastermix (Kapabiosystems), 100 nM probe (5′-FAM-TGTACTTGTCCCTGCACCTCC TTGA-BHQ1–3′), 160 nM forward-primer (5′-CTCTGCTGCATGTAATGAAATC-3′), 160 nM reverse-primer (5′-AAGCTCAAGAGCACGATTAC-3′) (Biolegio) with the following program on a CFX real-time machine (Biorad): step 1) 5 min. 95 °C, step 2) 10 s. 95 °C, step 3) 5 s. 70 °C, step 4) 10 s. 55 °C, plate read, ramp rate 2 °C per second, go to step 2 for 49 times. The limit of detection (LOD) was the lowest tested concentration where at least 95% of the replicate measurements were positive. The LOD for the PCR on faeces is 10 pg/μl and for the PCR on mucosa samples is 0.1 pg/μl. Values < 10 and < 0.1, respectively, were considered negative and taken as 0.0 for calculation purposes.

### PCV2 PCR

Quantitative PCR (qPCR) was performed on serum samples as described previously [10]. DNA was extracted from the sera using a commercial kit (Roche, Magnapure 96 with DNA/viral NA SV kit). PCV2 genomic DNA in each sample was quantified by polymerase chain reaction (PCR), using primers and a probe specific for PCV2-ORF1. The cycle number where specific fluorescence exceeded the threshold was correlated with the cycle numbers of a set of samples containing known amounts of a PCV2-ORF1-containing plasmid. Results were expressed as log_10_ copies/μl of extracted DNA (log_10_ c/μl). Values lower than 2.00 log_10_ c/μl were considered negative and were taken as 0.0 log_10_ c/μl for calculation purposes.

### Statistical analysis

The level of significance was set at 0.05 and all tests were two-sided. Statistical analyses were carried out using SAS (SAS Institute Inc. Cary NC, USA). Where applicable (ADWG, qPCR faeces and qPCR ileum mucosa), the validity of AN(C)OVA was routinely checked on the homogeneity of the variance and normality of the residuals by visual inspection of the residual plots.

#### Serology

Antibody titres were evaluated using descriptive statistics. The average values were plotted with the 95% confidence interval where no overlap indicates statistical significance.

#### Diarrhoea score

In this challenge model, specific clinical signs due to challenge usually become apparent in the third week after challenge. The diarrhoea scores between days 13 and 21 were statistically analysed by a cumulative logit model [11] accounting for the correlation in the repeated measurements using Generalized Estimating Equations (GEE) with *p*-values based on empirical standard error.

#### Weight gain post-challenge

Since the effect of PPE on weight gain is observed mainly in the third week after challenge, the ADWG in this period (days 13 to 20) was calculated and statistically analysed by Analysis of CoVariance (ANCOVA) using the weight at day 13 as covariate and using Tukey’s post-hoc test to compare groups.

#### qPCR L. intracellularis: Faeces and ileum mucosa

qPCR data from faeces and ileum mucosa samples were log_10_ transformed (after adding 1 to avoid zeros) and expressed in log_10_ pg DNA/μl. The average values were plotted with the 95% confidence interval where no overlap indicates statistical significance.

In addition, the AUC was calculated by the linear trapezoidal rule as a measure of total shedding over time. The AUC of the qPCR data of faeces, the faeces qPCR data on day 21 and the qPCR data of the ileum mucosa on day 21 were statistically analysed by Analysis of Variance (ANOVA) using Tukey’s post-hoc test to compare groups.

#### qPCR PCV2: serum

PCV2 qPCR data in serum samples were log_10_ transformed (after adding 1 to avoid zeros) and expressed in log_10_ copies/μl. The average values were plotted with the 95% confidence interval where no overlap indicates statistical significance.

In addition, the AUC was calculated by the linear trapezoidal rule as a measure of total viral load over time. Because the AUC data was zero-inflated (i.e. median, 1st and 3rd quartile were all 0), ANOVA is no longer an appropriate approach to use. The AUC data of serum were therefore statistically analysed by Wilcoxon Rank sum test. Besides this, the data were evaluated at animal level in the Fisher’s exact test where an animal was considered positive if one or more samples was positive.

#### Gross pathology and histology ileum

The macroscopic total ileum lesion score and the total histology score were statistically analysed by a cumulative logit model [11] with *p*-values based on Likelihood-Ratio. The odds ratio was defined here as the odds on having lower classes in the vaccine group relative to that in the control group.

#### Mortality rate in the field trial

The mortality was evaluated by a generalised linear mixed model for binomials using a logit link [11] with treatment as fixed effect.

## Results

### Study 1

**Fig. 1:**
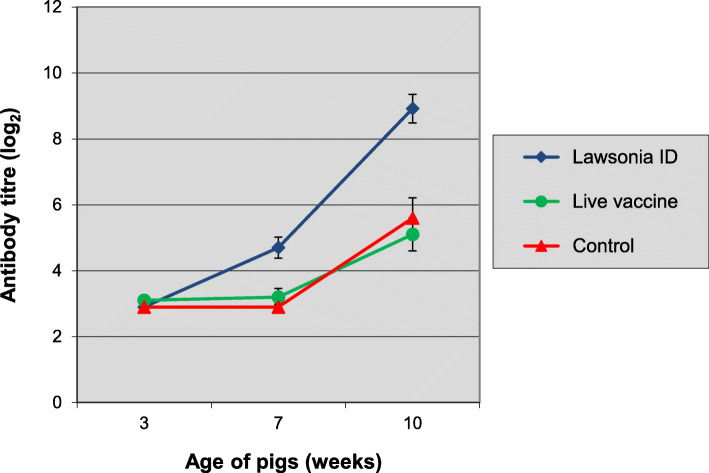
Time-course of anti-*L. intracellularis* antibody development in study 1. Group 1 was vaccinated at 3 weeks of age with Porcilis® Lawsonia ID, group 2 was vaccinated at 3 weeks of age with the live vaccine and group 3 was left unvaccinated. All pigs were challenged with *L. intracellularis* infected gut mucosa *at* 7 weeks of age. Bars indicate 95% confidence interval

On the day of vaccination, all pigs were either seronegative for *L. intracellularis* or had a very low antibody titre with an average titre of 3.1 log_2_ (Fig. 1). On the day of challenge (4 weeks after vaccination), active seroconversion was evident with a clear increase in antibody titre level in Porcilis® Lawsonia ID vaccinated group (avg titre 4.7 log_2_), whereas the antibody titres measured in the live vaccinated group and the control group remained comparable to the pre-vaccination levels. All groups had developed an increased antibody response by the day of necropsy, 3 weeks after challenge, with a higher antibody titre level in group 1 (avg titre 8.9 log_2_) compared to groups 2 (avg titre 5.1 log_2_) and 3 (avg titre 5.6 log_2_).

During the study, two animals were culled before the scheduled day of post-mortem examination. Twenty-four days after vaccination, one animal of group 2 was found dead without previous clinical signs. Necropsy, bacteriological and histological examination revealed a purulent meningitis caused by *Streptococcus suis* as the most likely cause of death. Another pig of group 3 was euthanized 1 day before challenge after days of increasing locomotory problems. Necropsy revealed that this pig had coronitis and malformation of the hip.

**Fig. 2:**
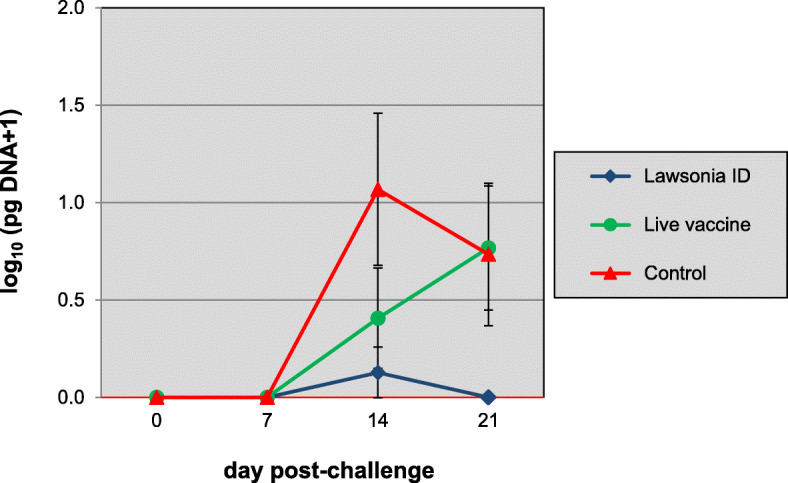
Time-course of shedding of *L. intracellularis* as determined in a qPCR on faeces. Group 1 was vaccinated at 3 weeks of age with Porcilis® Lawsonia ID, group 2 was vaccinated at 3 weeks of age with the live vaccine and group 3 was left unvaccinated. All pigs were challenged with *L. intracellularis* infected gut mucosa *at* 7 weeks of age. Bars indicate 95% confidence interval

Although clinical signs were hardly observed after challenge, the control animals developed a clear *L. intracellularis* infection characterized by, sub-optimal weight gain and bacterial fecal shedding (Table 2 and Fig. 2). Upon necropsy the *L. intracellularis* infection was confirmed by suggestive macroscopically visible ileum lesions (mucosal reddening and thickening), positive ileum mucosa PCR and immuno-histological ileum lesion scores. Pigs vaccinated with Porcilis® Lawsonia ID showed a statistically significantly better weight gain (i.e. ADWG 956 g vs 674 g) and a significantly reduced shedding (qPCR faeces), bacterial load (qPCR ileum mucosa) and macroscopic as well as microscopic ileum lesion scores when compared with the controls. In addition, all these parameters were also significantly better when compared to group 2 (live vaccine group). In this latter group, most of the parameters were also improved when compared to the controls, but the effects were less pronounced than those of group 1. The differences between group 2 and the controls were statistically significant only for total shedding and the microscopic ileum lesion scores.

**Table 2:** Post-challenge results ± SD of vaccination-challenge studies 1 and 2

vaccine group	avg clinical score day 13-21	ADWG g/day day 13-20	PCR faeces avg log pg DNA/μl		PCR mucosa avg log pgDNA/μl day 21	avg macroscopic ileum score day 21	avg microscopic ileum score (IHC) day 21
			AUC	day 21			
**Study 1: vaccination at 3 weeks of age, challenge at 7 weeks of age, necropsy 21 days after challenge**							
Law ID^a^	0.3 ± 0.5	956 ± 119^d,e^	0.13 ± 44^d,e^	0.0 ± 0.0^d,e^	0.03 ± 0.04^d,e^	0.6 ± 1.5^d,e^	0.1 ± 0.3^d,e^
Live vaccine^b^	0.2 ± 0.4	812 ± 287	0.79 ± 0.91^d^	0.77 ± 0.81	0.50 ± 0.51	61 ± 81	3.4 ± 3.2^d^
Control	0.5 ± 1.0	674 ± 381	1.44 ± 1.13	0.73 ± 0.93	0.66 ± 0.60	68 ± 125	5.7 ± 3.3
**Study 2: vaccination at 3 weeks of age, challenge at 24 weeks of age, necropsy 21 days after challenge**							
Law ID + PCV ID^c^	1.3 ± 1.9^d^	1001 ± 710^d^	4.23 ± 1.51	0.71 ± 0.96^d^	0.19 ± 43^d^	129 ± 165^d^	2.9 ± 2.8^d^
Control	3.8 ± 5.4	-139 ± 1210	5.02 ± 1.65	1.90 ± 1.08	0.54 ± 61	241 ± 160	7.7 ± 2.6

^a^Porcilis® Lawsonia ID

^b^commercially available live attenuated Lawsonia vaccine

^c^associated mixed use of Porcilis® Lawsonia ID and Porcilis® PCV ID

^d^*p*<0.05 vs control

^e^*p*<0.05 vs live vaccine

### Study 2

**Fig. 3:**
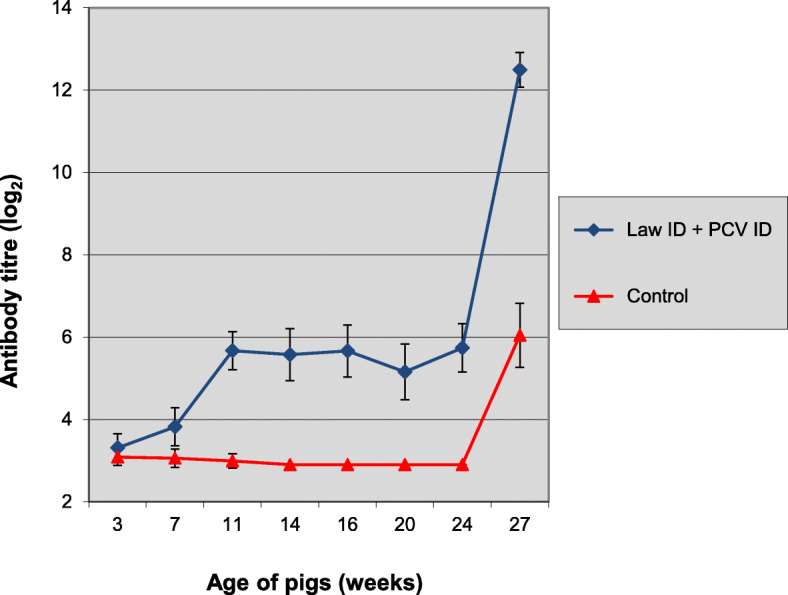
Time-course of anti-*L. intracellularis* antibody development in study 2. Group 1 was vaccinated at 3 weeks of age with Porcilis® Lawsonia ID in associated mixed use with Porcilis® PCV ID and group 2 was left unvaccinated. All pigs were challenged with *L. intracellularis* infected gut mucosa at 24 weeks of age. Bars indicate 95% confidence interval

On the day of vaccination all pigs were either seronegative for *L. intracellularis* or had a low antibody titre with an average titre of 3.2 log_2_ (Fig. 3). After vaccination, active seroconversion was evident with a clear increase in antibody titre level in the Porcilis® Lawsonia ID vaccinated group (avg titre up to 5.7 log_2_), whereas the antibody titres measured in the control group remained comparable to the pre-vaccination levels. Both groups showed an increased antibody response by the day of necropsy, 3 weeks after challenge, with a clearly higher antibody titre level in group 1 (avg titre 12.5 log_2_) compared to group 2 (avg titre 6.0 log_2_).

**Fig. 4:**
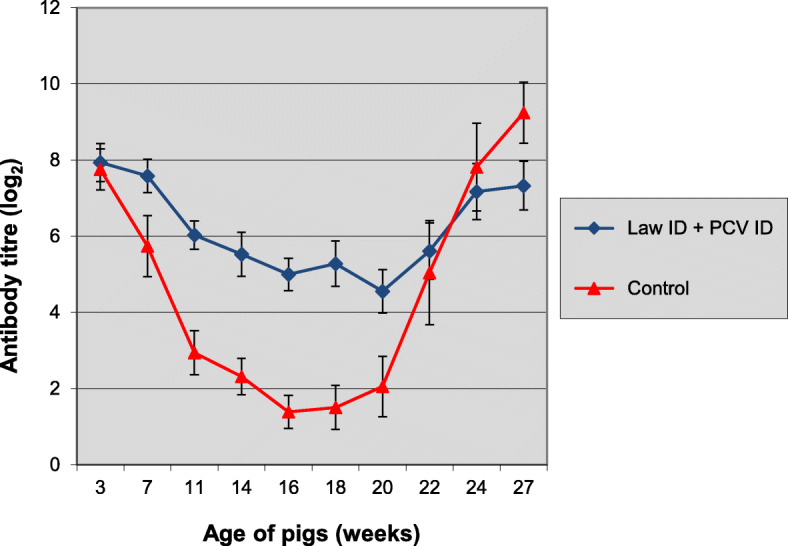
Time-course of anti-PCV2 antibody development in study 2. Group 1 was vaccinated at 3 weeks of age with Porcilis® Lawsonia ID in associated mixed use with Porcilis® PCV ID and group 2 was left unvaccinated. All pigs were challenged with *L. intracellularis* infected gut mucosa at 24 weeks of age. Bars indicate 95% confidence interval

On the day of vaccination all pigs had moderate to high maternally derived antibody titres against PCV2 (Fig. 4). The average titre of the control group showed a steady and clear decrease compared to day of vaccination until approximately 16–18 weeks of age after which a strong increase was observed, which is indicative for a field infection.

During the study three animals died or were culled before the scheduled day of post-mortem. One animal (group 2) was found dead on day 29 post-vaccination after having shown signs of depression. Necropsy and bacteriological examination revealed that the cause of death was related to a severe necrotizing enteritis, most likely caused by *Clostridium sordelii*, as this bacterium was predominantly and abundantly cultured from the lesion. Another pig (group 1) was found dead without previous signs on day 48 after vaccination. Necropsy revealed an intestinal torsion (duodenum) as the most likely cause of death. One pig of the control group (group 2) was euthanized 17 days after challenge on account of PPE specific disease i.e. several days of diarrhoea, weight loss and deteriorating condition. Necropsy showed that this pig had a severe enteritis with fibrin deposits in jejunum, ileum and colon. The presence of *L. intracellularis* was confirmed by PCR and IHC.

**Fig. 5:**
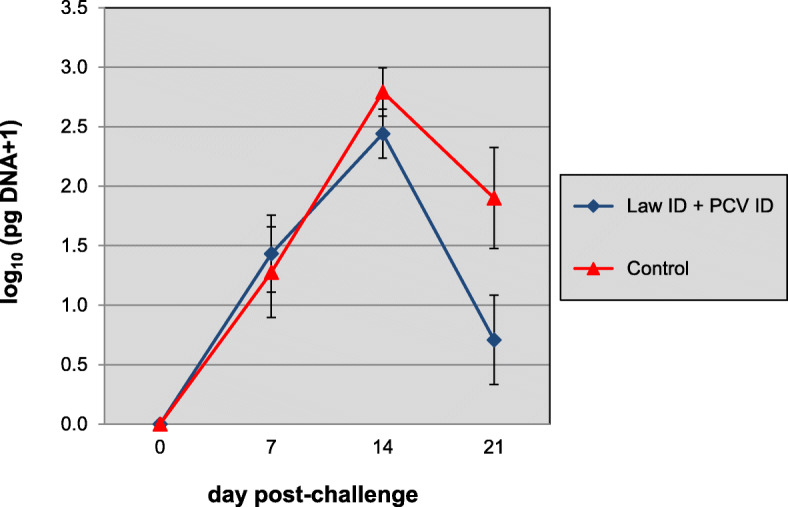
Time-course of shedding of *L. intracellularis* in faeces as determined by qPCR. Group 1 was vaccinated at 3 weeks of age with Porcilis® Lawsonia ID in associated mixed use with Porcilis® PCV ID and group 2 was left unvaccinated. All pigs were challenged with *L. intracellularis* infected gut mucosa at 24 weeks of age. Bars indicate 95% confidence interval

In the third week after challenge the control animals developed clinical signs of *L. intracellularis* characterised by diarrhoea, weight loss, and shedding of *L. intracellularis* in the faeces (Table 2 and Fig. 5). Upon necropsy the *L. intracellularis* infection was confirmed by typical macroscopically visible ileum lesions (mucosal reddening and thickening), positive mucosa PCR and IHC ileum lesion scores. Pigs vaccinated with Porcilis® Lawsonia ID showed a statistically significant reduction in clinical signs, *L. intracellularis* associated weight loss (i.e. ADWG 1001 g vs -139 g), *L. intracellularis* shedding (qPCR faeces day 21), bacterial load (qPCR ileum mucosa) and macroscopic as well as microscopic ileum lesion scores when compared to the controls.

**Fig. 6:**
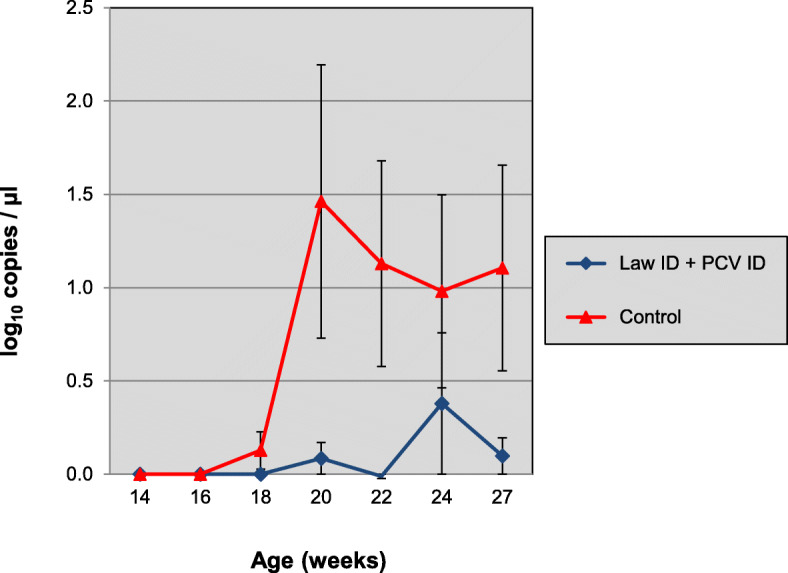
Time-course of PCV infection as determined by qPCR of sera samples. Group 1 was vaccinated at 3 weeks of age with Porcilis® Lawsonia ID in associated mixed use with Porcilis® PCV ID and group 2 was left unvaccinated. All pigs were challenged with *L. intracellularis* infected gut mucosa at 24 weeks of age. Bars indicate 95% confidence interval

Since the serological results indicated that a PCV field infection had occurred, the available serum samples of 14, 16, 18, 20, 22, 24 and 27 weeks of age were tested in a quantitative PCV2 PCR (Fig. 6). The AUC of the qPCR data was calculated as a measure of total viral load over time. Average AUC values were 1.26 and 9.64 log_10_ (copies/μl) x week, for vaccinates and controls, respectively, which was statistically significant (*p* < 0.0001, Wilcoxon Rank sum test). The proportion of pigs that were PCR positive were 5/24 in the vaccine group vs 18/24 in the control group. This difference also was statistically significant (*p* = 0.0004, Fisher’s exact test).

### Field trial

After the start of the study, the *L. intracellularis* associated mortality was 0.45% in the vaccine group compared to 1.65% in the control group. This difference was statistically significant (*p* = 0.0022). Also, the overall mortality was significantly lower in the vaccine group as compared to the control group (1.43% vs 2.63%, *p* = 0.0184). To support the results of the field trial, overall mortality, ADWG and feed conversion rate were derived from the farm data management system which contained data from study and non-study animals to compare historically. After the start of the study the overall mortality decreased form 2.4% (in the year preceding the study) to 2.1% during the study period. The ADWG gradually increased from 843 g/day in the year preceding the study to 859 g/day during the study, and the feed conversion rate (kg feed/kg body weight) decreased from 2.41 in the year preceding the study to 2.31 during the study.

## Discussion

Intradermal vaccination, using a needle free and intradermal injector such as the IDAL, improves animal health and food safety as there is no risk of needle breakage or transmission of disease by re-use of needles. The IDAL allows for a dose volume of 0.2 ml into the dermis which has the advantage of the presence of dendritic cells at the site of administration and the close proximity of skin-draining lymph nodes, resulting in a direct response to the antigen in the vaccine. Therefore, the adjuvant and/or antigen load may be lower to achieve a comparable or even better efficacy [12].

In a previous study we reported the efficacy of a novel vaccine for intramuscular use, Porcilis® Lawsonia [6]. This vaccine consists of a freeze-dried *L. intracellularis* antigen fraction that is dissolved in adjuvant just before intramuscular use. The freeze-dried nature of the antigen provides additional flexibility with regard to vaccination as it can also be dissolved in Porcilis PCV M Hyo (associated mixed use), an existing inactivated combination vaccine against porcine Circovirus type 2 and *Mycoplasma hyopneumoniae* and making it possible to vaccinate against 3 major swine pathogens with one single intramuscular injection.

A logical next step for the development of a new intradermal vaccine is to dissolve the same freeze-dried antigen fraction in an intradermal adjuvant (= Porcilis® Lawsonia ID). The intradermal adjuvant (X-Solve®) is identical to the adjuvant of Porcilis® PCV ID, an existing inactivated intradermal vaccine against porcine Circovirus type 2, making it possible to administer both vaccines in associated mixed use and to further extend the flexibility of vaccination against these major swine pathogens.

For the experimental challenge studies pigs from a farm with no history of PPE were used. On the day of vaccination nearly all pigs were seronegative, but a few pigs had low antibody titres. Although the ELISA has been validated and shown to be specific for *L. intracellularis,* the status of the low titres in a few animals is not clear. The fact that the low values decrease to sub-detectable in the course of time and the fact that the animals remain seronegative up to 24 weeks of age, suggest that the low titres were maternally derived and aspecific. Irrespective of the status of the low antibody titres, the pigs were PCR negative (faecal samples) before challenge and were susceptible to the *L. intracellularis* challenge at 7 weeks as well as at 24 weeks of age, allowing evaluation of vaccine efficacy.

In study 1, pigs vaccinated with Porcilis® Lawsonia ID showed a statistically significantly better weight gain and a significantly reduced shedding (qPCR faeces), bacterial load (qPCR ileum mucosa) and macroscopic as well as microscopic ileum lesion scores when compared with the controls. All these parameters were also significantly better when compared to the pigs vaccinated with the live vaccine. Given the nature of PPE, being a local intestinal infection, one would expect an attenuated live vaccine administered orally to be protective rather than an inactivated vaccine that is administered systemically. In this study we demonstrate however that an inactivated vaccine administered intradermally is highly efficacious against *L. intracellularis* infection which is in line with previous results of Jacobs et al. [6] and Roerink et al. [7] who also showed good protection against experimental *L. intracellularis* infection using inactivated whole cell vaccines. Although the exact protective mechanism is not known, and opposite to the general immunological understanding, the inactivated vaccine protected much better compared to a commercially available live vaccine.

In study 2 the efficacy of Porcilis® Lawsonia ID was tested in associated mixed use with Porcilis® PCV ID against challenge with *L. intracellularis* at 21 weeks after vaccination. This study was biased by a PCV2 field infection that occurred from 16 weeks of age onwards and with different impact on the two treatment groups as the controls (group 2) were not vaccinated against PCV2. On the other hand, a PCV2 field infection is a realistic scenario that will occur in field situations as well. The PCV2 infection could have exacerbated the clinical signs of the *L. intracellularis* challenge control group. Indeed, the impact of the challenge in study 2 was more severe if compared to study 1. The difference could also be due to the use of different challenge material or the age of the animals. It is the authors experience (unpublished) that when using the same challenge material, more severe clinical signs are induced in older animals (24 weeks of age) if compared to younger animals (7 weeks of age).

Regardless of the impact of the PCV2 field infection in study 2, the associated mixed-use vaccination of Porcilis® Lawsonia ID and Porcilis® PCV ID induced a statistically significantly better weight gain and significantly reduced clinical signs, *L. intracellularis* shedding (qPCR faeces), bacterial load (qPCR ileum mucosa) and macroscopic as well as microscopic ileum lesion scores. In addition, the associated mixed-use vaccination was also efficacious in reducing the PCV infection since the PCV2 viral load in serum samples was statistically significantly reduced in the vaccine group compared to the control group as determined by PCR.

The field study was performed on a farm with a history of mortality due to acute ileitis occurring a few weeks before slaughter. Economically, this is the worst-case scenario for a farmer since he has maximally invested in the animals by this point. After the start of the vaccinations, the *L. intracellularis* associated mortality and overall mortality showed a significant decrease in the vaccinated animals. In addition, although not statistically substantiated, the key production parameters (on farm level, including non-study animals) also improved during the course of the study when compared with the historic data: overall mortality (from 2.4 to 2.1%), ADWG (from 843 to 859 g/day) and feed conversion rate (from 2.41 kg to 2.31 kg feed/kg body weight). It should be kept in mind that these improvements are an underestimation of the real effect because they were calculated for the whole herd, whereas only part of the pigs were vaccinated. If the whole herd had been vaccinated, the improvement of the key production parameters would likely have been even better, not only by the direct effect of vaccination but also by additional indirect effects as described by Knight-Jones et al. [13]. These indirect effects may result from reduced shedding, as shown in the experimental studies, and subsequent reduced infectious pressure resulting in even greater positive effects when a whole herd is vaccinated.

## Conclusion

In this study the novel inactivated intradermal vaccine Porcilis® Lawsonia ID, either as standalone, or in associated mixed use with Porcilis® PCV ID, induced statistically significant protection against experimental *L. intracellularis* infection. This was demonstrated by lower clinical scores, improved weight gain, reduction of *L. intracellularis* shedding and reduction of macroscopic as well as microscopic ileum lesion scores, when compared to control animals. In the field trial, the vaccine provided reduction in *L. intracellularis* associated mortality.

## Data Availability

The datasets used and analysed during the current study are available from the corresponding author on reasonable request.
